# Development of a Chromosomally Integrated Metabolite-Inducible Leu3p-α-IPM “Off-On” Gene Switch

**DOI:** 10.1371/journal.pone.0012488

**Published:** 2010-08-31

**Authors:** Maria Poulou, Donald Bell, Kostas Bozonelos, Maria Alexiou, Anthony Gavalas, Robin Lovell-Badge, Eumorphia Remboutsika

**Affiliations:** 1 Stem Cell Biology Laboratory, Institute of Molecular Biology and Genetics, Biomedical Sciences Research Center “Alexander Fleming,” Attica, Greece; 2 Division of Stem Cell Biology and Developmental Genetics, MRC National Institute for Medical Research, London, United Kingdom; 3 Transgenics Unit, Institute of Immunology, Biomedical Sciences Research Center “Alexander Fleming,” Attica, Greece; 4 Division of Developmental Neurobiology, MRC National Institute for Medical Research, London, United Kingdom; Institute of Genetics and Molecular and Cellular Biology, France

## Abstract

**Background:**

Present technology uses mostly chimeric proteins as regulators and hormones or antibiotics as signals to induce spatial and temporal gene expression.

**Methodology/Principal Findings:**

Here, we show that a chromosomally integrated yeast ‘Leu3p-α-ΙΡΜ’ system constitutes a ligand-inducible regulatory “off-on” genetic switch with an extensively dynamic action area. We find that Leu3p acts as an active transcriptional repressor in the absence and as an activator in the presence of α-isopropylmalate (α-ΙΡΜ) in primary fibroblasts isolated from double transgenic mouse embryos bearing ubiquitously expressing Leu3p and a Leu3p regulated GFP reporter. In the absence of the branched amino acid biosynthetic pathway in animals, metabolically stable α-IPM presents an EC_50_ equal to 0.8837 mM and fast “OFF-ON” kinetics (t_50_ON = 43 min, t_50_OFF = 2.18 h), it enters the cells via passive diffusion, while it is non-toxic to mammalian cells and to fertilized mouse eggs cultured *ex vivo*.

**Conclusions/Significance:**

Our results demonstrate that the ‘Leu3p-α-ΙΡΜ’ constitutes a simpler and safer system for inducible gene expression in biomedical applications.

## Introduction

Temporal and spatial control of gene activity is a fundamental tool for regulated protein expression for basic, pharmaceutical and clinical research [1], [2], [3]. The most popular inducible systems use protein chimeras, antibiotics or hormones for induction and include the tetracycline system [1], the systems of the recombination enzyme Cre/loxP [2] and Flipase [3], the EcR (ecdysone) system [4] and the CRE-ER^T2^ system based on the ligand-binding domain of the estrogen receptor [5]. The “OFF/ON” gene switches allow for the expression of cytotoxic and dominant negative proteins [6], for the ability to reverse the expression of the target gene [7], for the study of “gain of function” and “loss of function phenotypes” [8] and for the ability to isolate protein targets of transcription factors [9]. However, drawbacks include the use of hormones and antibiotics as regulators of gene expression, which result in cytotoxicity and developmental defects in animal models, making it difficult to study the function of genes involved in embryonic development [10], the high cost of the inducer [11], leakiness in the absence of the inducer [12] and chromosomal alterations [13]. As a result, development of tools that allow for tighter control of gene induction with limited side effects are necessary for gene function analysis in animal models and safe clinical protocols for gene and stem cell therapy.

Leu3p belongs to the Zn(II)_2_-Cys_6_ cluster family[17], [18]. Leu3p is a pleiotropic transregulator with a molecular function resembling that of the thyroid hormone receptors (TR) [14], namely acting as an active repressor of transcription in the absence (“OFF”) and as an activator in the presence (“ON”) of its ligand, α-isopropylmalate (α-ΙΡΜ), a metabolic intermediate of the leucine biosynthetic pathway in yeast [15]. Leu3p binds with a high affinity (K_d_ = 3 nM) [16] to upstream promoter elements (UAS_LEU_) with a consensus everted repeat sequence 5′-GCCGGNNCCGGC-3′ [16] present in a number of genes involved in branched amino-acid biosynthesis in yeast [18], [21]. Leu3p consists of four domains, the zinc cluster DNA binding domain located in amino acids 37–67, resembling the Gal4 DNA binding domain [17], a linker region that specifies binding to the everted repeat target site [23], [24], an alpha-helix/heptad repeat domain from amino acids 85–102 involved in dimerization [14] middle region that is involved in the regulation of Leu3p activity (Ligand Binding Domain) by α-IPM [19], [23], [25] and finally an acidic activation domain from amino acids 856–886, self-masked in the absence of α-ΙΡΜ [19], [26], [27], [28]. The mechanisms used by Leu3p as a transcriptional regulator are conserved throughout plants and mammals [18] and could involve TBP [30], [31], [32]. Leu3p is able to transcribe genes solely and specifically in the presence of its effector molecule α-ΙΡΜ [17] in yeast, in transiently transfected mouse pre-adipocytes [18] and fibroblasts [19] as well as *in vitro* [15].

Here, we demonstrate that a chromosomally integrated “Leu3p-α-ΙΡΜ” can be used as a highly specific inducible gene expression system. Taking advantage of the fact that the leucine biosynthetic pathway exists only in prokaryotes, fungi and superior plants, but not in animals [20], we generated transgenic mice and found that the “Leu3p-α-IPM” system is a safe and efficient “OFF-ON” gene switch in double transgenic primary mouse embryo fibroblasts, thus paving the way for a number of applications in gene regulation studies and biomedicine.

## Results

### Generation of transgenic mice and primary mouse embryonic fibroblasts

**Figure 1:**
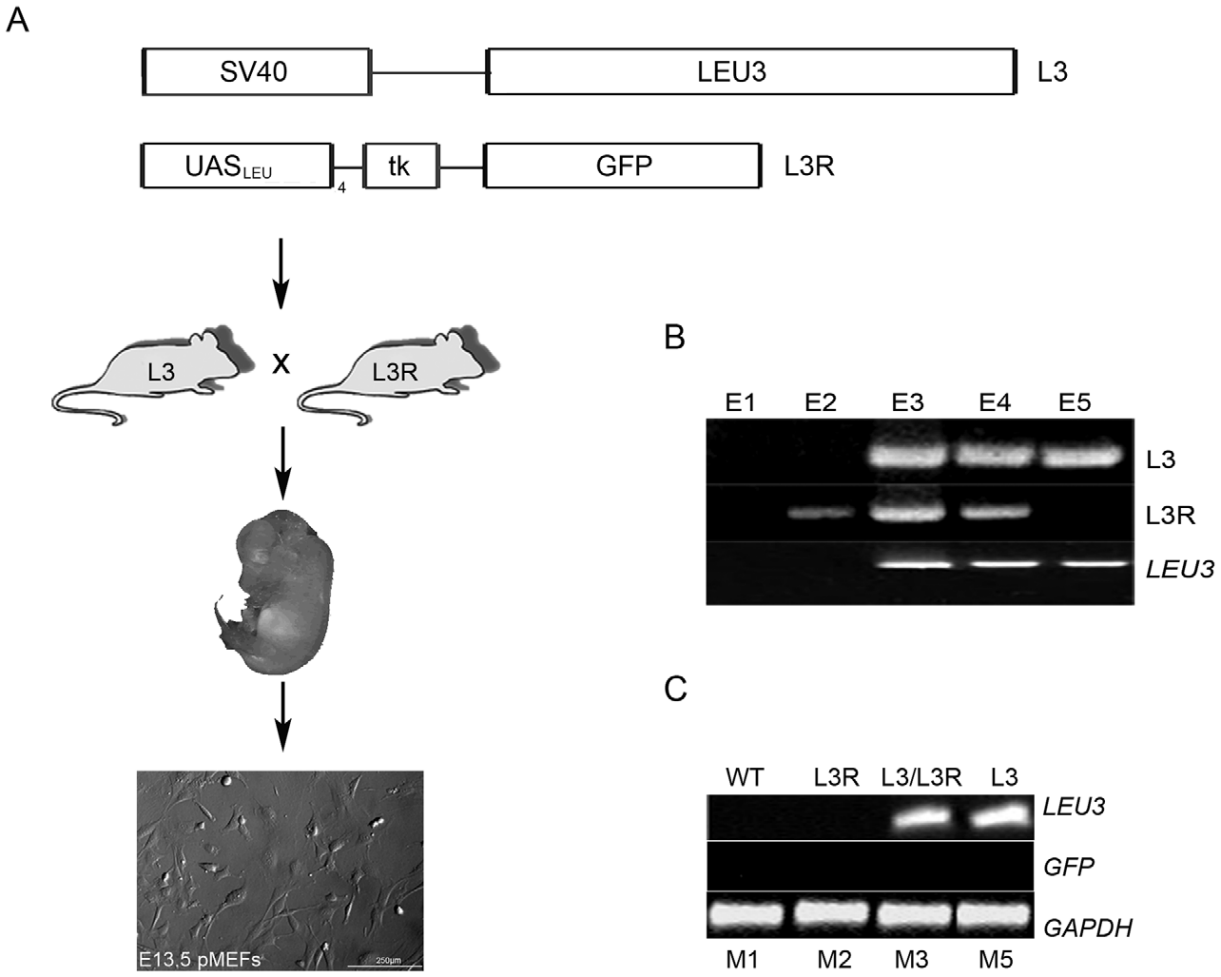
Generation of transgenic mice and primary mouse embryonic fibroblasts (pMEFs). (A) Top panel: scheme of the plasmid constructs used for the generation of the L3 and L3R transgenic mouse lines. Lower panel: scheme of the cross between the transgenic mouse lines and isolation of pMEFs from E13.5 mouse embryos. (B) PCR and RT-PCR analysis of mouse embryos showing Leu3 expression specifically in embryos expressing L3 transgene. (C) RT-PCR analysis of pMEFs showing Leu3 expression in the cell lines isolated from L3 transgenic embryos and lack of GFP expression in L3R pMEFs when α-ΙΡΜ is not added in the culture. RT-PCR for GAPDH was used as a control. L3: Leu3 transgene/L3R: Leu3 reporter transgene/E1-5: embryo 1/M1-5: pMEF cell line 1 (scale bar 200μm).

To assess whether the “Leu3p-α-IPM” expression system is functional in mice, we generated transgenic mice expressing Leu3p ubiquitously under the control of the SV40 promoter (L3). As a reporter, we have used GFP driven by four copies of the Leu3p-dependent upstream activating sequence (UAS_LEU_) enhancer positioned upstream of the thymidine kinase minimal promoter (L3R) (Figure 1A). Leu3p binding to UAS_LEU_ should actively repress the expression of GFP in the absence of α-IPM and activate transcription in the presence of α-IPM [15]. We obtained five transgenic lines expressing Leu3p ubiquitously (ER2a-e) and another eight expressing the reporter GFP under the control of Leu3p enhancer (ER4a-h) with variable degrees of germ line transmission (data not shown). Matings between the L3 and L3R lines were set up, α-IPM was administered intraperitonially at day E7.5 for 2 days in 12 h intervals, the embryos were harvested at E9.5 and E10.5 and assayed for fluorescence. None of the embryos showed detectable GFP fluorescence signal (data not shown). Thus, we could not assess, whether the GFP was not functional with regard to fluorescence or the system was not responding to α-IPM.

At that point, we decided to generate primary mouse fibroblasts (pMEFs) from double transgenic E13.5 embryos and assay for the function of the “Leu3p-α-IPM” system *ex vivo*. From timed L3 and L3R matings, two double transgenic embryos (E3 and E4), one wt embryo (E1), one embryo harboring the L3 transgene (E5) and one embryo harboring the L3R transgene were harvested (E2). The transgenes were identified using a polymerase chain reaction (PCR) for the presence of the L3 and L3R constructs (Figure 1B, L3 and L3R). To establish that the L3 transgene was indeed expressing the Leu3 mRNA, we isolated total RNA from the E1-E5 embryos and assayed for *LEU3* mRNA expression in RT-PCR experiments. This was evident in E3-E5 embryos that bore the L3 transgene, demonstrating that the *LEU3* mRNA was indeed expressed in those embryos (Figure 1B, *LEU3*).

Primary mouse embryonic fibroblasts (pMEFs) were then isolated from the E1-E5 embryo trunks and cultured *ex vivo* (M1-M5). As in the embryos, *LEU3* expression was also evident in the transgenic pMEFs isolated from L3 or L3/L3R transgenic embryos (Figure 1C, M1-M3, M5). To assess whether the “Leu3p-α-IPM” was leaky, we then assayed for *GFP* expression in pMEFs carrying the L3R transgene (M2 and M3). As anticipated [15], no GFP expression was detected in the absence of α-IPM (Figure 1C, *GFP*). Similar results were obtained with M4 pMEFs (data not shown). Thus, no leakiness was observed in the absence of α-IPM *in vivo* and *ex vivo* (Figure 1).

### “Leu3p-α-IPM” acts as an “OFF-ON” genetic switch in double transgenic primary mouse embryonic fibroblasts

**Figure 2:**
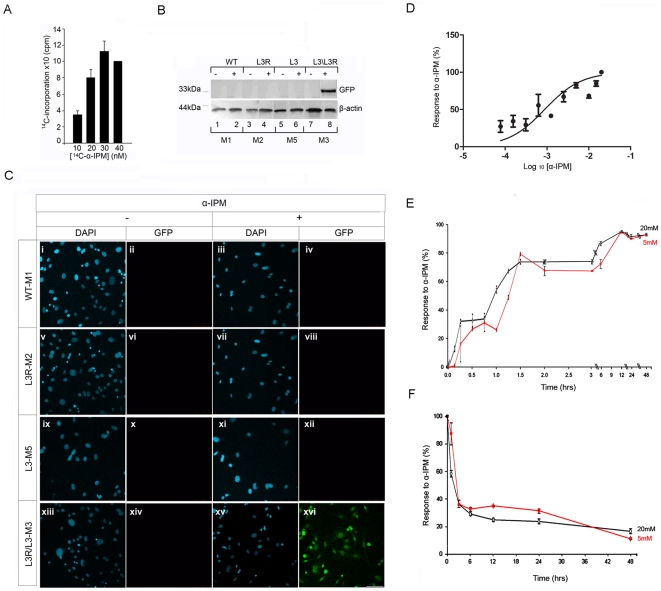
Analysis of Leu3p-α-ΙΡΜ inducible gene expression system in double transgenic primary mouse embryonic fibroblasts. (A) Incorporation of ^14^C-α-IPM into fibroblast cells. 10T1/2 were grown to confluency of 80–90% before they were incubated in the presence of a constant amount α-IPM (2 mM) and various amounts of ^14^C-α-IPM (10–40 nM). After 48 hours, the cells were lysed in the presence of digitonin and the radioactivity incorporated into the cell was counted. The average percent of ^14^C-α-IPM incorporated in the cells for each α-IPM concentration is presented as the mean ± standard deviation of the mean (SD) (Table S1). (B) and (C). *Ex vivo* analysis of “Leu3p-α-ΙΡΜ” inducible gene expression system in pMEFs. (B) Detection of GFP expression with western blot in primary fibroblasts in the presence or absence of α-ΙΡΜ. β-actin expression was used as a positive control. (C) Immunohistochemical detection of GFP expression in primary fibroblasts derived from the mating of L3 and L3R transgenic lines. GFP expression is detected only upon α-ΙΡΜ addition in the double transgenic fibroblasts. Results from GFP immunoreactivity analysis are in accordance with the results obtained from western blot. (D) Kinetics of α-ΙΡΜ. Titration of [α-ΙΡΜ] for maximum inducibility in primary mouse fibroblasts (pMEFs). WT, L3R and double transgenic pMEFS were cultured in the presence of increasing concentrations of α-ΙΡΜ (0, 0.078, 0.156, 0.312, 0.625., 1.25, 2.5, 5, 10, 15, 20 Mm) and induced GFP was quantitated. Following data analysis performed using the GraphPad PRISM 5 software (GraphPad, Inc., USA), the EC_50_ was calculated to be 0.8837 mM. The data are derived from three independent experiments for each experimental group (WT, L3R, L3/L3R) and for each different concentration of the inducer (0, 0.078, 0.156, 0.312, 0.625., 1.25, 2.5, 5, 10, 15, 20 Mm) and the absolute values are presented (Table S2) as the mean ± standard deviation of the mean (SD). (E) α-ΙΡΜ “ON” kinetics. Double transgenic pMEFS were cultured in the presence of 5 and 20 mΜ α-ΙΡΜ for different time points. The time required for 50% of inducible GFP expression is t_50_”ON” equal to 49±0.9 min after 5 mM α-IPM addition and to 43+3 min after 20 mM α-ΙΡΜ addition. (F) α-ΙΡΜ “OFF” kinetics. Double transgenic pMEFs were cultured in the presence of 5 and 20 mM α-ΙΡΜ for 24 hrs, then α-ΙΡΜ was removed from the media and cells were left in culture for a period up to 48 hrs. After α-IPM removal from the media, the time required for 50% reduction of GFP expression is t_50_OFF^5^ equal to 3.64±0.94 h, when the initial [α-IPM] concentration was 5 mM and t_50_OFF^20^ equal to 2.18±0.43 h, when the initial [α-IPM] concentration was 20 mM (scale bar: 50 μm). The data are derived from three independent experiments for each experimental group.

To assess the permeability of α-IPM, mouse fibroblasts were grown to confluency in the presence of variable amounts of ^14^C-α-ΙΡΜ supplemented with 2 mM non-radioactive α-IPM [16]. At the end of the 48 hr incubation period, the cells were lysed and the amount of ^14^C-α-ΙΡΜ incorporated into the cells was counted. The percentile of ^14^C-α-ΙΡΜ incorporation was found to be 0.28±0,039%, a value close to the theoretical one equal to 0.24% when equilibrium is established between a fibroblast cell and the milieu (Figure 2A; Table S1). Thus, we conclude that α-IPM is passively diffused into mammalian cells and as a result no additional yeast protein component is required for its entry into the cells.

To evaluate the function of “Leu3p-α-IPM” as a gene switch, we cultured wild type and transgenic pMEFs (M1-M3 and M5) for 12 hrs in the absence or presence of α-IPM at a final concentration of 20 mΜ (Figure 2B, C). GFP expression was detected using western blot (Figure 2B) and immunohistochemistry (Figure 2C) experiments in pMEFs isolated from wild type (M1), L3R (M2) and L3 (M5) transgenic embryos cultured either in the absence (Figure 2B, lanes 1, 3, 5 and 7 and Figure 2C, i-ii, v-vi, ix-x, xiii-xiv respectively) or presence of α-IPM (Figure 2B, lanes 2, 4, 6 and 8 and Figure 2C, iii-iv, vii-viii, xi-xii,xv-xvi respectively). In accordance to the role of Leu3p as an active transcriptional repressor [14], GFP expression was undetectable in pMEFs isolated from L3 and L3R double transgenic embryos (M3) when cultured in the absence of α-IPM (Figure 2B lane 7, Figure 2C, xiii-xiv). These observations, demonstrate that indeed “Leu3p-α-IPM” gene expression system is not leaky when its components are chromosomally integrated. However, GFP protein was detected in all M3 cells upon α-IPM induction, documented by GFP immunoreactivity using a specific anti-GFP antibody both in western blot (Figure 2B, lane 8) and immunofluorescence (Figure 2C, xv-xvi) experiments. These results demonstrate that the “Leu3p-α-IPM” is a tightly controlled gene expression system in double transgenic pMEFs.

To study the kinetics of α-IPM, we assayed for the concentration of α-ΙΡΜ required for optimal Leu3p-dependent induction of GFP expression in double transgenic pMEFs, cultured in the presence of increasing concentrations of α-ΙΡΜ (0.078, 0.156, 0.312, 0.625, 1.25, 2.5, 5, 10, 15, 20 mM) and GFP protein was detected and quantitated using indirect immunofluorescence (Figure 2D). As a baseline for the assay, we recorded the GFP expression in L3/L3R double transgenic pMEFs in the absence of α-IPM and as negative controls corresponding values from L3R and WT MEFs in the presence of increasing concentrations of α-ΙΡΜ (Table S2,). Consistent with previous observations obtained in Figure 2B and 2C, background fluorescence was recorded from WT, L3R treated with various concentrations of α-ΙΡΜ and L3/L3R pMEFS (Table S2). As expected, when α-IPM was added to the media of the double transgenic L3/L3R pMEFs, the response to increasing concentrations of α-IPM was recorded and the EC_50_ was calculated to be at 0.8837 mΜ (Figure 2D).

The time required for GFP induction was also analyzed in double transgenic pMEFs. Cells were cultured in the presence of either 5 or 20 mΜ α-ΙΡΜ for different time points from 5 min to 48 hrs. GFP expression was detected and quantitated using indirect immunofluorescence (Figure 2E). Induction of GFP reaches half of its maximum level (a) 49 min after 5 mM α-IPM addition (t_50_ON^5^ = 49±0.9 min) and (b) 43 min after 20 mM α-ΙΡΜ addition (t_50_ON^20^ = 43±3 min) and it increases proportionally until it reaches a plateau 12 hrs after the addition of the ligand.

Finally, the kinetics of reversibility after α-IPM withdrawal was assayed for a period of 48 hrs. Three hours after withdrawal of 5 mM α-ΙΡΜ, GFP levels fall down to 50% (t_50_OFF^5^ = 3.64±0.94 h), while it takes two hours for GFP levels to fall down to 50% after withdrawal of 20 mM α-ΙΡΜ (t_50_OFF^20^ = 2.18±0.43 h). GFP levels continue to drop within 48 hrs after withdrawal (Figure 2F). Conclusively, there is no need for an additional protein component or for specific receptors in order for α-IPM to enter into mammalian cells, as α-IPM diffuses passively into fibroblasts to specifically induce GFP expression with fast “ON/OFF” kinetics.

### α-IPM is not toxic to early mouse embryos

**Figure 3:**
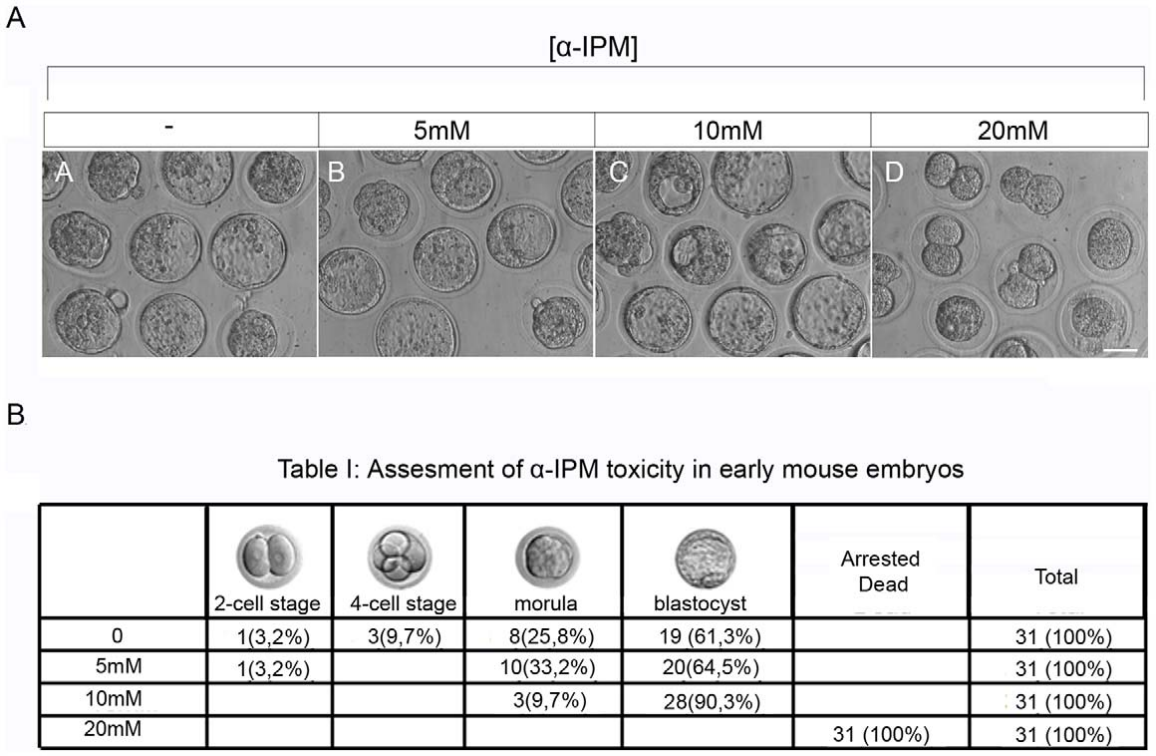
α-IPM toxicity in early mouse embryos. Two cell stage embryos were harvested from F1 pregnant females and cultured for two days *ex vivo* in the presence of increasing concentrations of α-IPM (0, 5, 10 or 20 mM) until they reach the blastocyst stage. Thirty one embryos were used for every experimental group. (A) Bright field photographs of embryos cultured for two days under different concentrations of α-ΙΡΜ. In the presence of α-ΙΡΜ in concentrations of 5 and 10 mM, two cell stage embryos develop normally to blastocyst stage compared to the control. However, early mouse embryos cultured in the presence of 20 mM α-ΙΡΜ arrest at the 2-cell stage due to osmolarity changes. (scale bar 50μm) (B) Assessment of α-IPM toxicity in early mouse embryos *ex vivo*.

The effects of α-IPM on primary fibroblasts were benign. As early embryos suffer from adverse effects of commonly used-inducers, such as tamoxifen [21] and tetracycline [22], it was important to explore any potential toxicity effects of α-IPM during embryonic development in pregnant females and early embryos in culture. Pregnant females were injected intraperitoneally on their seventh day of pregnancy with 25 mM of α-IPM. All embryos harvested at E11.5 were phenotypically normal, while mothers themselves did not exhibit any abnormal phenotypes (data not shown). Then, we assayed for the effects of α-IPM in early mouse embryos. Two cell stage embryos (124 embryos in total) were harvested and cultured in 4 groups for two days with 0, 5, 10 or 20 mM α-IPM (Figure 3A). In the absence of the inducer 61% of the embryos reached the blastocyst stage. When embryos were cultured in the presence of 5 or 10 mM of the inducer, similar numbers reached the blastocyst stage (Figure 3B); however, all embryos were arrested at the two-cell stage, when cultured at 20 mM α-ΙΡΜ, attributed to either the sensitivity of these embryonic stages to drastic changes in osmolarity [23] or possible toxicity effects created by the high levels of the inducer (Figure 3A and B). Therefore, α-IPM provides for a wide range of concentrations for inducibility with undetectable toxicity to pregnant females and early embryos *in vivo* and *ex vivo*.

## Discussion

Since the first transcriptional regulatory systems [24], several novel inducible gene expression systems were developed which have applications in gene function analysis [25], drug discovery [26], gene therapy [27] and engineering of desired phenotypes during development and in adult life [28]. An ideal regulatory system should be a) activated by a specific non-toxic bioavailable exogenous ligand, b) inactivated when the ligand is not present or removed from the media and c) its should not interfere with endogenous mammalian gene expression and metabolic pathways. We have developed a novel heterologous inducible gene expression system, based on a yeast transcription factor Leu3p, namely Leu3p-α-ΙΡΜ that meets these criteria.

α-IPM functions as an intermediate during leucine biosynthesis in yeast and activates specifically Leu3p-dependent transcription, both in vivo [29] and in vitro [15] and in mammalian cells [18] (Figure 2B,C). Compared to commonly used inducers tamoxifen [21] and tetracycline [22], [30] that can cause adverse effects during development, α-IPM is an ideal molecular matchmaker since it lacks toxicity (Figure 2 and 3), has metabolic stability [20] and lipid solubility (Figure 2A). The fact that α-IPM functions as an inducer of Leu3p activity in yeast extracts [17], in mouse pre-adipocytes [18], in mouse fibroblasts (Figure 2A) and in double transgenic pMEFs (Figure 2B–F) in a range of concentrations with no additional yeast component required for its function demonstrates that α-IPM can act as a safe highly specific ligand.

Another advantage of the “Leu3p-α-IPM” system is the tight regulation of Leu3p-inducible promoters by active repression exerted by Leu3p in the absence of α-IPM [17]. Leu3p can associate with the DNA in the absence (repressor form) and the presence (activator form) of α-IPM *in vivo*, but transcriptional activation is exerted only in the presence of α-IPM [18]. Thus, in the absence of α-IPM, regulated genes are suppressed; in the presence of α-IPM, they are activated, as we have also demonstrated in chromosomally integrated components of the system in primary mouse fibroblasts (Figure 2B and C). This is due to the high binding affinity of Leu3p to the UAS_LEU3p_ elements (K_d_ = 3 nM) [16] without the need for specific partners, a drawback for inducible gene expression systems, and involves a self-masking mechanism for the activation domain [19], [28]. Our results in transgenic pMEFs confirm that “Leu3p α-IPM” switch is not leaky (Figures 1B and C, 2B and C), in accordance with the function of Leu3p as a transcriptional repressor in the absence of α-ΙΡΜ [17], [18], [20], [22]. We also demonstated that removal of α-IPM from the culture of double transgenic primary mouse fibroblasts results in the repression of GFP expression with rapid kinetics compared to other inducible gene expression systems, such as tetracycline and FK506/rapamycin inducible systems with slow induction and reversibility kinetics [31]. This feature will allow us to use this system efficiently for time-dependent and tissue-specific expression of any protein of interest bypassing toxicity and early lethality events due to non-specific or leaky expression.

The fact that the leucine biosynthetic pathway is absent from mammals greatly facilitates the use of the “Leu3p-α-IPM” system as a general transcriptional ‘OFF-ON’ switch in animals. The fact that its components fail to evoke an immune response and it enables seamless integration into the regulatory and metabolic network of the target cell is very important since it demonstrates that the mechanisms of gene expression tangled with Leu3p are conserved throughout evolution from yeast to mammals [14]. Furthermore, beyond known components of the basal transcriptional machinery [30], [31], [32], the presence of other species-specific proteins is not necessary in order for “Leu3p-α-IPM” to activate gene expression, in contrast to Gal4:Gal80, in which galactose induces the release of Gal80 [32] and to ER-based HSP90:CreER^T2^ system, in which tamoxifen induces the dissociation of HSP90 promoting the nuclear translocation of CreER^T2^ protein [33]. Furthermore, there is no need for the generation of fusion proteins in order for “Leu3p-α-IPM” to be functional (37). Finally, “Leu3p-α-IPM” provides for a range of expression levels from no expression to high levels in response to the inducer that can support platforms for tissue- or target-specific interventions. Nevertheless, the need still exists for the discovery of analogues for α-IPM to be used in nM and μM concentrations to overcome adverse osmolarity and possible toxicity effects in high concentrations.

Overall, we show that “Leu3p-α-IPM” is a purely heterologous inducible regulatory “OFF/ON”gene switch with an extensive dynamic action area that provides specificity, lack of interference to known cellular pathways in animals, lack of toxicity, fast inducibility and reversibility, bioavailability and dose–dependence. These advantages pave the way for applications of the “Leu3p-α-IPM” gene switch for a wide range of developmental studies, inducible gene targeting and transgenesis in mice and other organisms, drug discovery, gene therapy and stem cell therapy.

## Materials and Methods

### DNA constructs

SV40-Leu3 expression construct (L3) was generated by cloning the Leu3 cDNA, as an EcoRI-BamHI fragment into the p513 vector (a gift from D. Metzger) from the pMSV-Leu3 vector (Guo, 1990). L3 transgenic construct was isolated as an XhoI-XbaI fragment. A (UAS_LEU_)_4_-tk-LUC plasmid was initially generated by the insertion of a 100 bp double-stranded oligonucleotide harboring four UAS_LEU_ sequences behind the thymidine kinase minimal promoter into the pTK luciferase vector (a gift from Vincent Giguere). The (UAS_LEU_)_4_-tk fragment was cloned behind the mmGFP5 cDNA in the pG1 vector (a gift from Darren Gilmour). (UAS_LEU_)_4_-tk-GFP (L3R) transgenic construct was isolated as a NotI-HindIII fragment.

### ^14^C-α-ΙΡΜ incorporation

Fibroblasts were incubated in the presence of a constant amount (2 mM) α-ΙΡΜ and variable amounts of ^14^C-α-ΙΡΜ (10, 20,30 and 40 nM) until they reach confluency. Cells were lysed and the incorporated radioactivity from the lysed cells was counted in a scintillation counter. For each experimental group with different amounts of ^14^C-α-ΙΡM, two different samples were recorded and the average percent of radioactivity incorporated in the cells are presented in Table S1.

### Transgenic mice

Linearized DNA (L3 and L3R) was microinjected in pronuclei of fertilized egg. For L3 construct five founders were generated (ER2a-e) (germline transmission 25–50%). For L3R construct eight founders were generated (ER4a-h) (germline transmission in ER4a-c and ER4g 20–50%). All animals were handled in strict accordance with good animal practice as defined by the Animals Act 160/03.05.1991 applicable in Greece, revised according to the 86/609/EEC/24.11.1986 EU directive regarding the proper care and use of laboratory animals and in accordance to the Hellenic License for Animal Experimentation at the BSRC” Alexander Fleming” (Prot. No. 767/28.02.07) issued after protocol approval by the Animal Research Committee of the BSRC “Alexander Fleming” (Prot. No. 2762/03.08.05).

### Isolation of primary mouse embryonic fibroblasts (pMEFs)

E13.5 mouse embryos from L3R male to L3 female intercrosses were harvested and dissected in DMEM media. Heads were used for RNA preparation. Internal organs were used for genotyping. Carcasses were washed twice in PBS, minced finely, pieces were dissociated for 10 min with rotation at 37°C using trypsin/EDTA solution, triturated and filtered through a 70 μm mesh. Cells were plated in 10 cm tissue culture dishes (1 cultured dish per embryo) in standard media.

### Genotyping

Transgenic mice, embryos and pMEFs were genotyped by PCR (T = 62°C).

L3 (ER2c):5′CGAGGAGAACCTATTTCTTACAGTACCA3′ (L3-1003F) and, 5′TGATAATCGAGTCATTAAGTCTGTAGCCC3′ (L3-1348R) (345 bp).

L3R (ER4a): GFP-45F 5′CTGGAGTTGTCCCAATTCTTGTTG 3′ (forward) and GFP-428R 5′GATGTTTCCGTCCTCCTTGAAATC3′ (reverse) (383 bp).

### RT-PCR

Total RNA was isolated with TriZol (Invitrogen) and RT-PCR was carried out using the Qiagen One-Step RT-PCR system. The following primers were used: for GAPDH (Τ = 57°C): 5′-CATCTCTGCCCCCTCTGCTG-3′ (forward) and 5′-CGACGCCTGCTTCACCACCT-3′ (reverse); for Leu3 (T = 60°C): 5′-CGAGGAGAACCTATTTCTTACAGTACCA-3′ (forward) and 5′- TGATAATCGAGTCATTAAGTCTGTAGCCC-3′ (reverse) for GFP (T = 62°C) : GFP-45F 5′CTGGAGTTGTCCCAATTCTTGTTG 3′ (forward) and GFP-428R 5′GATGTTTCCGTCCTCCTTGAAATC3′ (T = 62°C). The size of the amplified products was 440 bp, 345 bp and 383 bp respectively.

### α-IPM preparation

A 500 mM stock solution of (+)-2-α-isopropylmalic acid (α-IPM) (Sigma-Aldrich, Taufkirchen, Germany) was prepared in ddH_2_0 and the pH was adjusted to 7.0 with 10N KOH.

### Immunohistochemistry

Transgenic pMEFs were fixed in 4% paraformaldehyde in 0.12 M PB, pH 7.2 at 4°C for 5 min and incubated in blocking buffer (BB) (0. 12 M PB, pH 7.2, 0.15% glycine, 2 mg/ml BSA fragment V (Gibco-Invitrogen, Thessaloniki, Greece) and 0.1% Triton X-100) for 1 h on ice. Cells were incubated o/n at 4°C with an anti-GFP rabbit polyclonal antibody (Santa Cruz Biotechnology Inc., Heidelberg, Germany) in 1∶1000 dilution in BB. After extensive washes with PBS at RT, cells were incubated with secondary antibody (Alexa 488-conjugated anti-rabbit, 1∶500) (Molecular Probes-Invitrogen, Thessaloniki, Greece) for 1 h at RT. Samples were stored in anti-fade DAPI mounting media (Molecular Probes - Invitrogen, Thessaloniki, Greece).

### Western Blotting

Cells were harvested with trypsin, pellet was washed with PBS and dissolved in cold buffer A (20 mM Tris-HCl, 420 Mm NaCl, 0.2 Mm EDTA, 0.5 mM DTT, 25% glycerol, 0.5 mM PMSF, 1.5 mM MgCl2, 0.5% NP40) supplemented with Protease Inhibitor Cocktail (Sigma-Aldrich, Taufkirchen, Germany). Incubation at 4°C for 15 min and centrifuge for 15 min at 10,000×g followed. Protein concentration of the supernatant was determined by Bradford assay. Proteins (20μg per lane) were separated on 10% SDS-polyacrylamide gel, transferred to nitrocellulose membrane and membrane was blocked in western blot blocking buffer (5% milk, 10 mM Tris-HCl pH 7.6, 0.15 mM NaCl, 0.05% Tween-20) for 2 h at RT, incubated o/n with the primary antibody at 4°C. Goat anti-GFP polyclonal antibody (Santa Cruz Biotechnology, Inc, Santa Cruz, CA,U.S.A) and goat β-actin polyclonal antibody (Cell Signaling Technology, Inc, Danvers, MA,USA) were used (1∶1000). After extensive washing in TBST.1 (10 mM Tris-HCl, 0.15 mM NaCl, 0.1% Tween-20), goat anti-rabbit HRP conjugated secondary antibody was applied (1∶10,000) for 2 h at RT. Proteins were visualized by chemiluminescence detection using ECL (Cell Signaling Technology, Inc., Danvers, MA,USA).

### α-IPM toxicity effects in pregnant mice and early mouse embryos

α-IPM (25 mM) was injected into pregnant females intraperitonially at the seventh day of pregnancy and embryos were harvested at E11.5. No obvious abnormalities were detected. To assay α-IPM toxicity effects in early mouse embryos, fertilized eggs in the two-cell stage where harvested from pregnant F1 females and incubated in KSOM media with variable concentrations of α-IPM (0–20 mM) *ex vivo* for two days until they reach the blastocyst stage.

### Quantitation of GFP protein

The levels of induced GFP protein after indirect immunofluorescence using an anti-GFP antibody were quantified in double transgenic pMEFs in a Fluorescence plate reader TECAN Infinite M200 (wavelength range of 488 nm–522 nm).

## Supporting Information

Table S1 Average percent of 14C-α-ΙΡΜ incorporated in fibroblast cells.
(0.03 MB DOC)

Table S2 Response to increasing α-ΙΡΜ concentrations recorded from wt, L3R and L3/L3R pMEFs.
(0.05 MB DOC)
