# Supplementary material for: Development of a Chromosomally Integrated Metabolite-Inducible Leu3p-α-IPM “Off-On” Gene Switch
Source: PLoS One. 2010 Aug 31;5(8):e12488. doi: 10.1371/journal.pone.0012488 (PMC2930855; doi:10.1371/journal.pone.0012488)
Supplement: Table S1 — Average percent of 14C-α-ΙΡΜ incorporated in fibroblast cells. (0.03 MB DOC) [file pone.0012488.s001.doc]

**Table S1**: Average percent of ^14^C-α-ΙΡΜ incorporated in fibroblast cells

| **[^14^C-α-ΙΡΜ]**  **(nM)** | **Radioactivity incorporated**  **(% + SD)** |
| --- | --- |
| 10 | 0,31±0,007 |
| 20 | 0,33±0,035 |
| 30 | 0,305±0,017 |
| 40 | 0,205±0,0035 |
