# Supplementary material for: Development of a Chromosomally Integrated Metabolite-Inducible Leu3p-α-IPM “Off-On” Gene Switch
Source: PLoS One. 2010 Aug 31;5(8):e12488. doi: 10.1371/journal.pone.0012488 (PMC2930855; doi:10.1371/journal.pone.0012488)
Supplement: Table S2 — Response to increasing α-ΙΡΜ concentrations recorded from wt, L3R and L3/L3R pMEFs. (0.05 MB DOC) [file pone.0012488.s002.doc]

**Table S2**: Response to increasing α-ΙΡΜ concentrations recorded from wt, L3R and L3/L3R pMEFs.

| **[α-ΙΡΜ]**  **(mM)** | **WT**  **(Fluorescence units+SD)** | **L3R**  **(Fluorescence units+SD)** | **L3/L3R**  **(Fluorescence units+SD)** | **ΔL3/L3R ^[x-0]^**  **(Fluorescence units+SD^Δ^)** |
| --- | --- | --- | --- | --- |
| 0 | 8902±515 | 9067±731 | 11658±1063 | 0 |
| 0.078 | 7901±720 | 8815±541 | 14383±476 | 2725± 672 |
| 0.156 | 8646±443 | 7383±1041 | 16810±525 | 5152±684 |
| 0.312 | 8654±446 | 7680±1515 | 18974±480 | 7316±673 |
| 0.625 | 8414±279 | 8194±1436 | 18840±1441 | 7182±1033 |
| 1.25 | 8296±670 | 8245±502 | 20518±2067 | 8860±1341 |
| 2.5 | 7169±583 | 6693±1297 | 23097±2926 | 11439±1797 |
| 5 | 8414±617 | 8405±1032 | 24730±1434 | 13072±1030 |
| 10 | 7471±264 | 6693±1670 | 24468±1752 | 12810±1183 |
| 15 | N/A | N/A | 25209±967 | 13551±830 |
| 20 | 8815±541 | 8405±348 | 28400±171 | 16742±622 |

N/A: non-treated
